# Editorial: What can we make of theories of embodiment and the role of the human mirror neuron system? An enduring, ever larger question

**DOI:** 10.3389/fnhum.2023.1229084

**Published:** 2023-06-13

**Authors:** Analía Arévalo, Fernando González-Perilli, Juliana V. Baldo, Agustin Ibáñez, Adolfo García

**Affiliations:** ^1^Division of Functional Neurosurgery, University of São Paulo Medical School, São Paulo, Brazil; ^2^Facultad de Información y Comunicación, Departamento de Medios y Lenguajes, Universidad de la República, Montevideo, Uruguay; ^3^VA Northern California Health Care System, Martinez, CA, United States; ^4^Centro de Neurociencias Cognitivas, Universidad de San Andres, Buenos Aires, Argentina; ^5^School of Psychology, Trinity College Dublin, Dublin, Ireland; ^6^National Scientific and Technical Research Council (CONICET), Buenos Aires, Argentina; ^7^Latin American Institute for Brain Health, Adolfo Ibáñez University, Santiago, Chile; ^8^Global Brain Health Institute, San Francisco, CA, United States; ^9^Trinity College Dublin, Dublin, Ireland; ^10^Departamento de Lingüística y Literatura, Facultad de Humanidades, Universidad de Santiago, Santiago, Chile

**Keywords:** embodiment, mirror neurons, mirror neuron system, human mirror neurons, embodied cognition

In 2013, 43 authors came together to produce a collection of 14 articles aiming to answer the following question: What can we make of theories of embodiment and the role of the human mirror neuron system? A decade later, this topic continues to engage researchers and clinicians alike, and, as is often the case in science, still fuels controversies and raises new and exciting questions. Therein lies the motivation for this new Research Topic, in which new ideas and techniques are integrated to further the conversation.

First, Kemmerer invites us to revisit the relation between syntax, action, and left BA44, discussing two competing hypotheses. One hypothesis suggests that the very same neural mechanisms in left BA44 subserve hierarchical sequencing for syntax and action; the other hypothesis suggests that hierarchical sequencing within these domains is subserved by anatomically distinct but functionally parallel neural mechanisms in left BA44. Kemmerer emphasizes that neither hypothesis has significantly more explanatory power than the other, and he delves deep into the last several years of imaging and electrophysiological data, as well as linguistic and evolutionary theories, to help us understand the evidence supporting both ideas.

Second, Trasmundi and Toro highlight an interesting link between mind wandering and reading. The authors contest the widely accepted view that mind wandering is detrimental for reading flow, comprehension, and the capacity to make inferences based on a text. By integrating embodied accounts of mind wandering and reading, they suggest that reading is multi-actional and benefits from drawing on different cognitive strategies spanning mind wandering processes and goal-oriented behavior. They propose that rather than being pernicious, mind wandering may enrich cognitive processes underlying reading, such as imagining and reflection. This article offers a new perspective on the cognitive processes of mind wandering as well as reading, and presents a potential new strategy for more effective reading. In other words, mind wandering may now be considered a positive rather than negative influence on reading and may also be studied in the context of other similarly-demanding cognitive tasks.

Third, Visani et al. use behavioral and MEG data to examine the extent to which the processing of action pictures and words share common neural mechanisms, as classic embodiment theories contend. The authors report comparable engagement of the sensorimotor system by both types of stimuli, supporting specific theories of embodied semantics. By leveraging the high spatio-temporal resolution of MEG, this work fruitfully complements the larger corpus of evidence from temporally imprecise techniques, such as fMRI.

Fourth, Johari et al. apply high definition tDCS to stimulate the left hand motor area (HMA) and anterior inferior parietal lobe (aIPL) in healthy controls using an action semantic task with three levels of semantic processing (subliminal, implicit and explicit). The authors report that stimulating HMA and aIPL exerts a facilitatory effect on action-related language processing (vs. non-action language processing), extending previous relevant studies showing a relationship between motor and language processing. Furthermore, they propose that the HMA plays a general role in the semantics of actions and manipulable objects, while the aIPL plays an important role when visuo-motor coordination is required.

Finally, previous studies showed that suppression (asynchronous firing and reduction of mu and beta rhythm oscillations) over sensorimotor regions occurs during both motor imagery and action observation of biological motion. In Grazia et al., the authors observe that combining two tasks involving both motor imagery and action observation resulted in different patterns of event-related synchronization and desynchronization, compared to tasks involving motor imagery alone. The authors discuss the implications of such findings for informing the design of motor training tasks for patients with movement disorders, such as Parkinson's disease.

Taken together, these contributions underscore the intimate and profuse links between language embodiment and motor brain systems. Current results support and challenge relevant models, prompting new questions and inspiring novel avenues to tackle those questions. We hope this second edition of our Research Topic brings new insights to old questions and contributes to the deepening of our understanding of the mechanisms underlying embodied cognition and their implications for everyday life.

## Author contributions

AA and AG wrote the editorial. All authors commented on and approved the final version. All authors contributed to the idea of the special topic, as well as reviewed and edited the submissions.

